# Chlorido{[(*E*)-2-(diphenyl­phosphan­yl)benzyl­idene](furan-2-ylmeth­yl)amine-κ*P*}gold(I)

**DOI:** 10.1107/S1600536812050404

**Published:** 2012-12-15

**Authors:** Haleden Chiririwa, Wade L. Davis

**Affiliations:** a Department of Chemistry, University of Cape Town, Private Bag, Rondebosch 7707, South Africa; b Research Centre for Synthesis and Catalysis, Department of Chemistry, University of Johannesburg (APK Campus), PO Box 524, Auckland Park, Johannesburg, 2006, South Africa

## Abstract

In the title complex, [AuCl(C_24_H_20_NOP)], the ligand has N, P and O electron-donating atoms but the Au^I^ atom is coordinated only by the ‘soft’ P atom and an additional Cl atom in an almost linear fashion. Important geometrical parameters include Au—P = 2.2321 (13) Å, Au—Cl = 2.2820 (13) Å and P—Au—Cl = 176.49 (5)°. The furan ring is disordered over two positions in a 0.51 (2):0.49 (2) ratio.

## Related literature

For general background to the title compound, see: Shaw (1999 ▶); Barnard *et al.* (2004 ▶); Nomiya *et al.* (2003 ▶). For the synthesis of the starting materials, see: Mogorosi *et al.* (2011 ▶); Uson & Laguna (1986 ▶). For similar compounds, see: Chiririwa & Muller (2012 ▶); Williams *et al.* (2007 ▶). For their applications, see: Chiririwa *et al.* (2013 ▶).
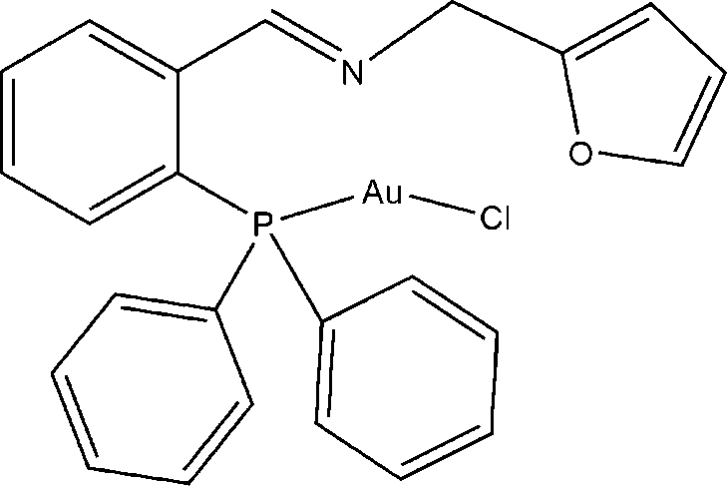


## Experimental

### 

#### Crystal data

[AuCl(C_24_H_20_NOP)]
*M* *_r_* = 601.80
Monoclinic, 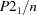

*a* = 13.4559 (4) Å
*b* = 10.3917 (2) Å
*c* = 17.2641 (4) Å
β = 111.751 (1)°
*V* = 2242.16 (9) Å^3^
*Z* = 4
Mo *K*α radiation
μ = 6.77 mm^−1^
*T* = 173 K
0.16 × 0.11 × 0.02 mm

#### Data collection

Bruker APEXII 4K CCD diffractometer
Absorption correction: multi-scan (*SADABS*; Bruker, 2007 ▶) *T*_min_ = 0.411, *T*_max_ = 0.877
74340 measured reflections
5536 independent reflections
4175 reflections with *I* > 2σ(*I*)
*R*_int_ = 0.100

#### Refinement

*R*[*F* ^2^ > 2σ(*F* ^2^)] = 0.039
*wR*(*F* ^2^) = 0.100
*S* = 1.07
5536 reflections
299 parameters
240 restraints
H-atom parameters constrained
Δρ_max_ = 2.27 e Å^−3^
Δρ_min_ = −1.52 e Å^−3^

### 

Data collection: *APEX2* (Bruker, 2007 ▶); cell refinement: *SAINT* (Bruker, 2007 ▶); data reduction: *SAINT* and *XPREP* (Bruker, 2007 ▶); program(s) used to solve structure: *SHELXS97* (Sheldrick, 2008 ▶); program(s) used to refine structure: *SHELXL97* (Sheldrick, 2008 ▶); molecular graphics: *DIAMOND* (Brandenburg & Putz, 2005 ▶); software used to prepare material for publication: *WinGX* (Farrugia, 2012) ▶.

## Supplementary Material

Crystal structure: contains datablock(s) I, global. DOI: 10.1107/S1600536812050404/yk2081sup1.cif

Structure factors: contains datablock(s) I. DOI: 10.1107/S1600536812050404/yk2081Isup2.hkl

Additional supplementary materials:  crystallographic information; 3D view; checkCIF report

## Figures and Tables

**Fig. 1.**
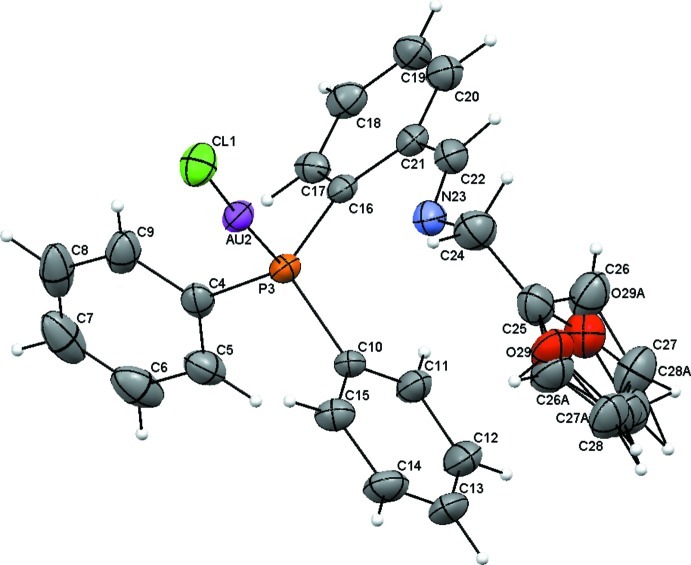
View of [Au(C24H20NOP)Cl] showing the atom labelling scheme and displacement ellipsoids drawn at the 40% probability level.

## supplementary crystallographic information

### Comment

There is a growing interest in the co-ordination chemistry of ligands containing both hard (N donor) and soft (P donor) Lewis bases. Such ligands have the potential to bind to soft metal centers such as those of the platinum group metals strongly *via* phosphorus and weakly *via* nitrogen, which allows for the displacement of the chelating N-moiety. This is very desirable in homogenous catalytic reactions and the catalytic application of P—N based ligands is being thoroughly investigated by our group.

Among the 'hard' donor type atoms, the co-ordination chemistry of gold(I) shows a distinct paucity in the literature. In this scenario the potentially bidentate ligand is chelated to the metal through only the phosphorus atom (Fig. 1). The gold complex showed a closely linear P— Au—Cl system (bond angle of 176.49°). Another important geometrical parameter includes the C22—N23 = 1.254 (6) Å which is consistent with C=N double bonding. The Au—P bond distance of 2.2321 (13) Å agrees with that reported by Williams *et al.*.

### Experimental

To a dry CH_2_Cl_2_ (10 ml) solution of the precursor [Au(tht)Cl] (tht = tetrahydrothiophene) was added an equimolar amount of *N*-{(*E*)-[2-(diphenylphosphanyl)phenyl]methylidene}-2-furan-2-ylethanamine in CH_2_Cl_2_ (10 ml), and stirred at room temperature for 2 hrs. The solvent was reduced under reduced pressure and on addition of hexane, the product was filtered off and washed with Et_2_O (2 *X* 5 ml)and dried under vacuum for 4 hrs affording a yellow precipitate. Crystals suitable for X-ray structure determination were obtained by recrystallization from a CH_2_Cl_2_-hexane mixture at room temperature.

### Refinement

The methine and aromatic H atoms were placed in geometrically idealized positions and constrained to ride on their parent atoms, with with C—H = 0.95 Å and *U*_iso_(H) = 1.2*U*_eq_(C) for aromatic, C—H = 0.99 Å and *U*_iso_(H) = 1.2*U*_eq_(C) for CH_2_ C—H = 0.95 Å and *U*_iso_(H) = 1.2*U*_eq_(C) for CH. A disorder refinement model was applied to the furyl ring in the asymmetric unit. Geometrical (*FLAT*) restaraints were applied to keep the ring planar.Bond distance (*DFIX*) and distance similarity restraints (*SADI*) were applied to obtain reasonable geometries. Ellipsoid displacement (*SIMU* and *DELU*) restraints were also applied to the disordered moiety. Free variables were connected to the disordered component to add to unity.

### Crystal data

**Table:**

[AuCl(C_24_H_20_NOP)]	*F*(000) = 1160
*M**_r_* = 601.80	*D*_x_ = 1.783 Mg m^−^^3^
Monoclinic, *P*2_1_/*n*	Mo *K*α radiation, λ = 0.71073 Å
Hall symbol: -P 2yn	Cell parameters from 5534 reflections
*a* = 13.4559 (4) Å	θ = 3.6–28.3°
*b* = 10.3917 (2) Å	µ = 6.77 mm^−^^1^
*c* = 17.2641 (4) Å	*T* = 173 K
β = 111.751 (1)°	Plate, yellow
*V* = 2242.16 (9) Å^3^	0.16 × 0.11 × 0.02 mm
*Z* = 4	

### Data collection

**Table:**

Bruker APEXII 4K CCD diffractometer	5536 independent reflections
Radiation source: fine-focus sealed tube	4175 reflections with *I* > 2σ(*I*)
Graphite monochromator	*R*_int_ = 0.100
Detector resolution: 0 pixels mm^-1^	θ_max_ = 28.3°, θ_min_ = 3.1°
0.5° ω scans, 20s	*h* = −17→17
Absorption correction: multi-scan (*SADABS*; Bruker, 2007)	*k* = −13→13
*T*_min_ = 0.411, *T*_max_ = 0.877	*l* = −23→23
74340 measured reflections	

### Refinement

**Table:**

Refinement on *F*^2^	Primary atom site location: structure-invariant direct methods
Least-squares matrix: full	Secondary atom site location: difference Fourier map
*R*[*F*^2^ > 2σ(*F*^2^)] = 0.039	Hydrogen site location: inferred from neighbouring sites
*wR*(*F*^2^) = 0.100	H-atom parameters constrained
*S* = 1.07	*w* = 1/[σ^2^(*F*_o_^2^) + (0.0574*P*)^2^] where *P* = (*F*_o_^2^ + 2*F*_c_^2^)/3
5536 reflections	(Δ/σ)_max_ = 0.003
299 parameters	Δρ_max_ = 2.27 e Å^−^^3^
240 restraints	Δρ_min_ = −1.52 e Å^−^^3^

### Special details

**Table:**

Geometry. All e.s.d.'s (except the e.s.d. in the dihedral angle between two l.s. planes) are estimated using the full covariance matrix. The cell e.s.d.'s are taken into account individually in the estimation of e.s.d.'s in distances, angles and torsion angles; correlations between e.s.d.'s in cell parameters are only used when they are defined by crystal symmetry. An approximate (isotropic) treatment of cell e.s.d.'s is used for estimating e.s.d.'s involving l.s. planes.
Refinement. Refinement of *F*^2^ against ALL reflections. The weighted *R*-factor *wR* and goodness of fit *S* are based on *F*^2^, conventional *R*-factors *R* are based on *F*, with *F* set to zero for negative *F*^2^. The threshold expression of *F*^2^ > σ(*F*^2^) is used only for calculating *R*-factors(gt) *etc*. and is not relevant to the choice of reflections for refinement. *R*-factors based on *F*^2^ are statistically about twice as large as those based on *F*, and *R*- factors based on ALL data will be even larger.

### Fractional atomic coordinates and isotropic or equivalent isotropic displacement parameters (Å^2^)

**Table:**

	*x*	*y*	*z*	*U*_iso_*/*U*_eq_	Occ. (<1)
Cl1	0.67118 (12)	−0.07954 (12)	0.82704 (9)	0.0504 (3)	
Au2	0.537496 (15)	0.068557 (17)	0.801052 (11)	0.03605 (9)	
P3	0.40775 (10)	0.21597 (12)	0.76820 (7)	0.0330 (3)	
C4	0.3211 (4)	0.1963 (5)	0.8271 (3)	0.0402 (11)	
C5	0.2133 (4)	0.2276 (6)	0.7948 (4)	0.0525 (14)	
H5	0.1813	0.2570	0.7389	0.063*	
C6	0.1517 (6)	0.2161 (7)	0.8440 (5)	0.0707 (19)	
H6	0.0775	0.2361	0.8213	0.085*	
C7	0.1992 (7)	0.1753 (6)	0.9262 (5)	0.075 (2)	
H7	0.1576	0.1680	0.9600	0.090*	
C8	0.3059 (7)	0.1456 (6)	0.9584 (4)	0.0696 (19)	
H8	0.3384	0.1187	1.0148	0.084*	
C9	0.3670 (5)	0.1546 (5)	0.9092 (3)	0.0543 (14)	
H9	0.4406	0.1320	0.9318	0.065*	
C10	0.3191 (4)	0.2085 (5)	0.6596 (3)	0.0342 (10)	
C11	0.2655 (4)	0.3144 (5)	0.6159 (3)	0.0477 (13)	
H11	0.2723	0.3954	0.6429	0.057*	
C12	0.2017 (5)	0.3020 (6)	0.5326 (4)	0.0626 (17)	
H12	0.1644	0.3750	0.5026	0.075*	
C13	0.1913 (5)	0.1858 (6)	0.4923 (3)	0.0588 (16)	
H13	0.1469	0.1788	0.4350	0.071*	
C14	0.2447 (5)	0.0805 (6)	0.5347 (4)	0.0561 (16)	
H14	0.2395	0.0006	0.5068	0.067*	
C15	0.3072 (4)	0.0910 (5)	0.6195 (3)	0.0436 (12)	
H15	0.3418	0.0169	0.6499	0.052*	
C16	0.4594 (4)	0.3787 (5)	0.7951 (3)	0.0326 (10)	
C17	0.4287 (4)	0.4506 (4)	0.8500 (3)	0.0373 (11)	
H17	0.3743	0.4178	0.8672	0.045*	
C18	0.4745 (5)	0.5690 (4)	0.8810 (4)	0.0463 (13)	
H18	0.4517	0.6157	0.9187	0.056*	
C19	0.5530 (5)	0.6175 (5)	0.8564 (4)	0.0522 (14)	
H19	0.5860	0.6974	0.8778	0.063*	
C20	0.5835 (5)	0.5500 (5)	0.8009 (4)	0.0499 (14)	
H20	0.6372	0.5856	0.7839	0.060*	
C21	0.5394 (4)	0.4311 (4)	0.7681 (3)	0.0392 (11)	
C22	0.5774 (4)	0.3688 (6)	0.7093 (3)	0.0458 (12)	
H22	0.6342	0.4075	0.6977	0.055*	
N23	0.5382 (3)	0.2656 (4)	0.6732 (3)	0.0441 (10)	
C24	0.5800 (5)	0.2119 (7)	0.6128 (4)	0.0604 (16)	
H24A	0.5925	0.1184	0.6225	0.072*	
H24B	0.6490	0.2533	0.6197	0.072*	
C25	0.5007 (5)	0.2348 (6)	0.5259 (4)	0.0577 (15)	
C26	0.478 (2)	0.354 (2)	0.5111 (14)	0.069 (4)	0.51 (2)
H26	0.5032	0.4279	0.5455	0.082*	0.51 (2)
C27	0.4039 (18)	0.344 (2)	0.4285 (13)	0.077 (4)	0.51 (2)
H27	0.3607	0.4145	0.3999	0.092*	0.51 (2)
C28	0.399 (2)	0.235 (2)	0.3948 (16)	0.075 (5)	0.51 (2)
H28	0.3521	0.2068	0.3413	0.090*	0.51 (2)
O29	0.4960 (14)	0.151 (2)	0.4681 (13)	0.062 (4)	0.51 (2)
C28A	0.3720 (18)	0.3008 (19)	0.4016 (13)	0.058 (4)	0.49 (2)
H28A	0.3158	0.3466	0.3607	0.070*	0.49 (2)
C27A	0.418 (2)	0.1871 (19)	0.3925 (15)	0.066 (4)	0.49 (2)
H27A	0.4114	0.1472	0.3413	0.079*	0.49 (2)
C26A	0.466 (3)	0.150 (5)	0.457 (3)	0.070 (5)	0.49 (2)
H26A	0.4823	0.0607	0.4651	0.084*	0.49 (2)
O29A	0.4309 (12)	0.3359 (15)	0.4912 (11)	0.072 (4)	0.49 (2)

### Atomic displacement parameters (Å^2^)

**Table:**

	*U*^11^	*U*^22^	*U*^33^	*U*^12^	*U*^13^	*U*^23^
Cl1	0.0522 (8)	0.0505 (8)	0.0493 (8)	0.0131 (6)	0.0198 (7)	0.0090 (6)
Au2	0.03944 (13)	0.03606 (13)	0.02786 (12)	0.00058 (8)	0.00691 (8)	0.00152 (8)
P3	0.0335 (6)	0.0360 (7)	0.0249 (6)	−0.0020 (5)	0.0057 (5)	0.0002 (5)
C4	0.054 (3)	0.036 (3)	0.033 (3)	−0.013 (2)	0.019 (2)	−0.007 (2)
C5	0.048 (3)	0.062 (4)	0.053 (3)	−0.019 (3)	0.025 (3)	−0.016 (3)
C6	0.074 (4)	0.075 (4)	0.079 (5)	−0.032 (4)	0.047 (4)	−0.038 (4)
C7	0.108 (5)	0.067 (4)	0.082 (5)	−0.039 (4)	0.073 (5)	−0.034 (4)
C8	0.128 (6)	0.049 (4)	0.048 (4)	−0.021 (4)	0.051 (4)	−0.010 (3)
C9	0.081 (4)	0.048 (3)	0.036 (3)	−0.009 (3)	0.024 (3)	−0.003 (2)
C10	0.032 (2)	0.039 (3)	0.026 (2)	−0.011 (2)	0.0051 (19)	−0.001 (2)
C11	0.054 (3)	0.044 (3)	0.033 (3)	−0.008 (2)	0.002 (2)	0.001 (2)
C12	0.059 (4)	0.064 (4)	0.044 (3)	−0.012 (3)	−0.005 (3)	0.011 (3)
C13	0.054 (3)	0.080 (4)	0.030 (3)	−0.021 (3)	0.000 (3)	−0.002 (3)
C14	0.053 (4)	0.066 (4)	0.040 (3)	−0.014 (3)	0.007 (3)	−0.017 (3)
C15	0.041 (3)	0.045 (3)	0.040 (3)	−0.004 (2)	0.009 (2)	−0.007 (2)
C16	0.030 (2)	0.036 (2)	0.025 (2)	0.000 (2)	0.0021 (19)	0.002 (2)
C17	0.033 (3)	0.033 (3)	0.041 (3)	−0.0011 (19)	0.008 (2)	−0.002 (2)
C18	0.052 (3)	0.039 (3)	0.041 (3)	0.004 (2)	0.009 (3)	−0.004 (2)
C19	0.062 (4)	0.036 (3)	0.047 (3)	−0.011 (3)	0.007 (3)	−0.003 (3)
C20	0.053 (3)	0.046 (3)	0.043 (3)	−0.007 (2)	0.008 (3)	0.002 (2)
C21	0.039 (3)	0.044 (3)	0.028 (2)	−0.004 (2)	0.004 (2)	0.005 (2)
C22	0.042 (3)	0.056 (3)	0.038 (3)	−0.010 (3)	0.014 (2)	0.002 (3)
N23	0.040 (2)	0.057 (3)	0.036 (2)	−0.007 (2)	0.015 (2)	0.003 (2)
C24	0.067 (4)	0.066 (4)	0.049 (3)	−0.013 (3)	0.022 (3)	−0.012 (3)
C25	0.070 (4)	0.062 (4)	0.046 (3)	−0.017 (3)	0.027 (3)	−0.005 (3)
C26	0.088 (10)	0.069 (9)	0.044 (8)	0.000 (9)	0.020 (8)	0.002 (7)
C27	0.091 (9)	0.090 (9)	0.048 (9)	0.006 (8)	0.025 (8)	0.007 (8)
C28	0.089 (9)	0.080 (10)	0.045 (7)	−0.008 (9)	0.012 (7)	0.000 (9)
O29	0.082 (9)	0.059 (5)	0.038 (7)	−0.008 (8)	0.016 (7)	−0.011 (5)
C28A	0.075 (8)	0.061 (9)	0.044 (8)	−0.003 (7)	0.026 (6)	0.003 (7)
C27A	0.092 (9)	0.069 (9)	0.036 (6)	−0.011 (8)	0.022 (6)	−0.005 (7)
C26A	0.084 (11)	0.064 (8)	0.044 (8)	−0.013 (9)	0.005 (8)	−0.004 (7)
O29A	0.077 (8)	0.076 (7)	0.063 (8)	−0.008 (6)	0.026 (7)	0.010 (6)

### Geometric parameters (Å, º)

**Table:**

Cl1—Au2	2.2820 (13)	C17—H17	0.9500
Au2—P3	2.2321 (13)	C18—C19	1.371 (8)
P3—C10	1.813 (5)	C18—H18	0.9500
P3—C4	1.821 (5)	C19—C20	1.368 (8)
P3—C16	1.822 (5)	C19—H19	0.9500
C4—C5	1.386 (7)	C20—C21	1.397 (7)
C4—C9	1.389 (7)	C20—H20	0.9500
C5—C6	1.394 (8)	C21—C22	1.446 (8)
C5—H5	0.9500	C22—N23	1.254 (6)
C6—C7	1.390 (10)	C22—H22	0.9500
C6—H6	0.9500	N23—C24	1.467 (7)
C7—C8	1.370 (10)	C24—C25	1.503 (9)
C7—H7	0.9500	C24—H24A	0.9900
C8—C9	1.386 (8)	C24—H24B	0.9900
C8—H8	0.9500	C25—C26	1.28 (2)
C9—H9	0.9500	C25—O29	1.30 (2)
C10—C11	1.377 (7)	C25—O29A	1.388 (17)
C10—C15	1.384 (7)	C25—C26A	1.41 (4)
C11—C12	1.381 (7)	C26—C27	1.41 (3)
C11—H11	0.9500	C26—H26	0.9500
C12—C13	1.374 (8)	C27—C28	1.27 (3)
C12—H12	0.9500	C27—H27	0.9500
C13—C14	1.364 (8)	C28—O29	1.68 (4)
C13—H13	0.9500	C28—H28	0.9500
C14—C15	1.396 (7)	C28A—C27A	1.37 (3)
C14—H14	0.9500	C28A—O29A	1.50 (2)
C15—H15	0.9500	C28A—H28A	0.9500
C16—C17	1.385 (7)	C27A—C26A	1.13 (5)
C16—C21	1.430 (7)	C27A—H27A	0.9500
C17—C18	1.391 (6)	C26A—H26A	0.9500
			
P3—Au2—Cl1	176.49 (5)	C19—C18—H18	120.4
C10—P3—C4	105.1 (2)	C17—C18—H18	120.4
C10—P3—C16	110.2 (2)	C20—C19—C18	119.6 (5)
C4—P3—C16	103.0 (2)	C20—C19—H19	120.2
C10—P3—Au2	112.74 (17)	C18—C19—H19	120.2
C4—P3—Au2	112.53 (18)	C19—C20—C21	123.1 (6)
C16—P3—Au2	112.55 (15)	C19—C20—H20	118.5
C5—C4—C9	119.1 (5)	C21—C20—H20	118.5
C5—C4—P3	122.7 (4)	C20—C21—C16	117.5 (5)
C9—C4—P3	118.1 (4)	C20—C21—C22	118.3 (5)
C4—C5—C6	120.3 (6)	C16—C21—C22	124.2 (4)
C4—C5—H5	119.8	N23—C22—C21	122.6 (5)
C6—C5—H5	119.8	N23—C22—H22	118.7
C7—C6—C5	119.7 (7)	C21—C22—H22	118.7
C7—C6—H6	120.1	C22—N23—C24	118.5 (5)
C5—C6—H6	120.1	N23—C24—C25	109.4 (5)
C8—C7—C6	120.0 (6)	N23—C24—H24A	109.8
C8—C7—H7	120.0	C25—C24—H24A	109.8
C6—C7—H7	120.0	N23—C24—H24B	109.8
C7—C8—C9	120.4 (6)	C25—C24—H24B	109.8
C7—C8—H8	119.8	H24A—C24—H24B	108.2
C9—C8—H8	119.8	C26—C25—O29	123.5 (16)
C8—C9—C4	120.4 (6)	O29—C25—O29A	109.9 (13)
C8—C9—H9	119.8	C26—C25—C26A	117 (2)
C4—C9—H9	119.8	O29A—C25—C26A	98.6 (19)
C11—C10—C15	119.4 (5)	C26—C25—C24	112.4 (12)
C11—C10—P3	122.9 (4)	O29—C25—C24	118.2 (11)
C15—C10—P3	117.7 (4)	O29A—C25—C24	131.8 (9)
C10—C11—C12	119.6 (5)	C26A—C25—C24	129.2 (18)
C10—C11—H11	120.2	C25—C26—C27	98.9 (17)
C12—C11—H11	120.2	C25—C26—H26	130.5
C13—C12—C11	121.1 (6)	C27—C26—H26	130.5
C13—C12—H12	119.5	C28—C27—C26	115 (2)
C11—C12—H12	119.5	C28—C27—H27	122.3
C14—C13—C12	119.9 (5)	C26—C27—H27	122.3
C14—C13—H13	120.1	C27—C28—O29	102.9 (19)
C12—C13—H13	120.1	C27—C28—H28	128.6
C13—C14—C15	119.6 (5)	O29—C28—H28	128.6
C13—C14—H14	120.2	C25—O29—C28	92.1 (17)
C15—C14—H14	120.2	C27A—C28A—O29A	103.8 (18)
C10—C15—C14	120.4 (5)	C27A—C28A—H28A	128.1
C10—C15—H15	119.8	O29A—C28A—H28A	128.1
C14—C15—H15	119.8	C26A—C27A—C28A	107 (3)
C17—C16—C21	118.0 (4)	C26A—C27A—H27A	126.3
C17—C16—P3	119.5 (4)	C28A—C27A—H27A	126.3
C21—C16—P3	122.2 (4)	C27A—C26A—C25	120 (4)
C16—C17—C18	122.6 (5)	C27A—C26A—H26A	119.8
C16—C17—H17	118.7	C25—C26A—H26A	119.8
C18—C17—H17	118.7	C25—O29A—C28A	106.0 (13)
C19—C18—C17	119.2 (5)		
			
C10—P3—C4—C5	−25.0 (5)	C18—C19—C20—C21	−1.0 (9)
C16—P3—C4—C5	90.5 (5)	C19—C20—C21—C16	−0.6 (8)
Au2—P3—C4—C5	−148.1 (4)	C19—C20—C21—C22	179.6 (5)
C10—P3—C4—C9	158.5 (4)	C17—C16—C21—C20	1.8 (7)
C16—P3—C4—C9	−86.0 (4)	P3—C16—C21—C20	−171.8 (4)
Au2—P3—C4—C9	35.4 (4)	C17—C16—C21—C22	−178.3 (5)
C9—C4—C5—C6	−0.6 (8)	P3—C16—C21—C22	8.0 (7)
P3—C4—C5—C6	−177.1 (4)	C20—C21—C22—N23	−175.9 (5)
C4—C5—C6—C7	1.2 (9)	C16—C21—C22—N23	4.3 (8)
C5—C6—C7—C8	−0.5 (9)	C21—C22—N23—C24	178.2 (5)
C6—C7—C8—C9	−0.8 (9)	C22—N23—C24—C25	−105.0 (6)
C7—C8—C9—C4	1.3 (9)	N23—C24—C25—C26	56.6 (14)
C5—C4—C9—C8	−0.6 (8)	N23—C24—C25—O29	−149.6 (13)
P3—C4—C9—C8	176.0 (4)	N23—C24—C25—O29A	34.5 (12)
C4—P3—C10—C11	85.8 (5)	N23—C24—C25—C26A	−136 (2)
C16—P3—C10—C11	−24.6 (5)	O29—C25—C26—C27	28 (2)
Au2—P3—C10—C11	−151.3 (4)	O29A—C25—C26—C27	−38 (3)
C4—P3—C10—C15	−95.4 (4)	C26A—C25—C26—C27	12 (3)
C16—P3—C10—C15	154.3 (4)	C24—C25—C26—C27	−179.4 (11)
Au2—P3—C10—C15	27.6 (4)	C25—C26—C27—C28	−11 (3)
C15—C10—C11—C12	−0.8 (8)	C26—C27—C28—O29	−4 (3)
P3—C10—C11—C12	178.1 (4)	C26—C25—O29—C28	−29 (2)
C10—C11—C12—C13	−0.3 (9)	O29A—C25—O29—C28	−2.8 (17)
C11—C12—C13—C14	−0.2 (10)	C26A—C25—O29—C28	42 (11)
C12—C13—C14—C15	1.9 (9)	C24—C25—O29—C28	−179.6 (11)
C11—C10—C15—C14	2.4 (8)	C27—C28—O29—C25	17 (2)
P3—C10—C15—C14	−176.5 (4)	O29A—C28A—C27A—C26A	15 (3)
C13—C14—C15—C10	−3.0 (9)	C28A—C27A—C26A—C25	−22 (5)
C10—P3—C16—C17	112.4 (4)	C26—C25—C26A—C27A	−1 (5)
C4—P3—C16—C17	0.7 (4)	O29—C25—C26A—C27A	−119 (13)
Au2—P3—C16—C17	−120.8 (4)	O29A—C25—C26A—C27A	19 (4)
C10—P3—C16—C21	−74.0 (4)	C24—C25—C26A—C27A	−168 (3)
C4—P3—C16—C21	174.3 (4)	C26—C25—O29A—C28A	130 (4)
Au2—P3—C16—C21	52.8 (4)	O29—C25—O29A—C28A	4.6 (16)
C21—C16—C17—C18	−1.7 (7)	C26A—C25—O29A—C28A	−6 (2)
P3—C16—C17—C18	172.1 (4)	C24—C25—O29A—C28A	−179.3 (9)
C16—C17—C18—C19	0.2 (8)	C27A—C28A—O29A—C25	−3.5 (18)
C17—C18—C19—C20	1.2 (8)		
